# Stereotactic body radiation therapy for locally advanced pancreatic cancer

**DOI:** 10.1371/journal.pone.0214970

**Published:** 2019-04-12

**Authors:** Jinhong Jung, Sang Min Yoon, Jin-hong Park, Dong-Wan Seo, Sang Soo Lee, Myung-Hwan Kim, Sung Koo Lee, Do Hyun Park, Tae Jun Song, Baek-Yeol Ryoo, Heung-Moon Chang, Kyu-pyo Kim, Changhoon Yoo, Jae Ho Jeong, Song Cheol Kim, Dae Wook Hwang, Jae Hoon Lee, Ki Byung Song, Yoon Young Jo, Jongmoo Park, Jong Hoon Kim

**Affiliations:** 1 Department of Radiation Oncology, Asan Medical Center, University of Ulsan College of Medicine, Seoul, Republic of Korea; 2 Department of Gastroenterology, Asan Medical Center, University of Ulsan College of Medicine, Seoul, Republic of Korea; 3 Department of Oncology, Asan Medical Center, University of Ulsan College of Medicine, Seoul, Republic of Korea; 4 Department of Surgery, Asan Medical Center, University of Ulsan College of Medicine, Seoul, Republic of Korea; 5 Department of Radiation Oncology, Kyungpook National University Chilgok Hospital, Daegu, Republic of Korea; University of Texas MD Anderson Cancer Center, UNITED STATES

## Abstract

**Purpose:**

Stereotactic body radiation therapy (SBRT) is a promising treatment modality for locally advanced pancreatic cancer (LAPC). We evaluated the clinical outcomes of SBRT in patients with LAPC.

**Patients and methods:**

We retrospectively analyzed the medical records of patients with LAPC who underwent SBRT at our institution between April 2011 and July 2016. Fiducial markers were implanted using endoscopic ultrasound guidance one week prior to 4-dimensional computed tomography (CT) simulation and daily cone beam CT was used for image guidance. Patients received volumetric modulated arc therapy or intensity modulated radiotherapy using respiratory gating technique. A median dose of 28 Gy (range, 24–36 Gy) was given over four consecutive fractions delivered within one week. Survival outcomes including freedom from local disease progression (FFLP), progression-free survival (PFS), and overall survival (OS) were analyzed. Acute and late toxicities related to SBRT were assessed.

**Results:**

A total of 95 patients with LAPC were analyzed, 52 of which (54.7%) had pancreatic head cancers. Most (94.7%) had received gemcitabine-based chemotherapy. The 1-year FFLP rate was 80.1%. Median OS and PFS were 16.7 months and 10.2 months, respectively; the 1-year OS and PFS rates were 67.4% and 42.9%, respectively. Among 79 patients who experienced failure, the sites of first failures were isolated local progressions in 12 patients (15.2%), distant metastasis in 55 patients (69.6%), and both in 12 patients (15.2%). Seven patients (7.4%) were able to undergo surgical resection after SBRT and four had margin-negative resections. Three patients (3.2%) had grade 3 nausea/vomiting during SBRT, and late grade 3 toxicity was observed in another three patients.

**Conclusions:**

LAPC patients who received chemotherapy and SBRT had favorable FFLP and OS with minimal treatment-related toxicity. The most common pattern of failure was distant metastasis, which warrants further studies on the optimal scheme of chemotherapy and SBRT.

## Introduction

Recent advances in radiotherapy techniques, including four-dimensional image acquisition, image-guided treatment, and respiratory-gated delivery as well as better understanding of normal organ tolerance to radiation have enabled delivery of high-dose radiation to tumors while minimizing the radiation dose to normal organs. Stereotactic body radiation therapy (SBRT) is a form of short course radiotherapy that allows conformal and accurate delivery of high doses of radiation. SBRT has demonstrated high rate of local control in patients with lung cancer or other malignancies [1,2].

In patients with locally advanced pancreatic cancer (LAPC) that are defined as surgically unresectable but have no evidence of distant metastasis (DM), DM is the usual course of progression [3]. Therefore, chemotherapy is used as first-line therapy for systemic control, and local treatment modalities such as radiotherapy are relegated to the next step. Nevertheless, local tumor control is important issue, therefore, the current standard care for LAPC patients includes a combination of chemotherapy and radiotherapy [4]. However, after conventional chemoradiotherapy (CRT), approximately a half of patients experience local progression that led to development of pancreatic pain, obstruction symptoms, and other morbidities that decrease their quality of life [5]. Indeed, conventional radiotherapy with concurrent chemotherapy usually requires 6–7 weeks to complete and carries the risk of acute and late toxicity that may delay or interrupt further intensive chemotherapy [6]. Thus, development of a more effective local treatment modality with short treatment duration is needed.

SBRT is a promising treatment modality for LAPC due to its short treatment duration and proven efficacy. Prior studies on SBRT for patients with LAPC have shown local control rates ranging from 70 to 100% [7–16]. However, some studies using single fraction SBRT reported considerable acute and late toxicities [7,12,14]. Subsequent studies have used fractionated SBRT scheme to reduce the side effects, but the optimal radiation dose and schedule have yet to be determined [10,11,13,15,16]. In the present study, we evaluated the clinical outcomes of SBRT using a respiratory-gated volumetric-modulated arc therapy (VMAT) or intensity modulated radiotherapy (IMRT) technique for patients with LAPC at a single center.

## Materials and methods

### Patient selection

We reviewed the medical records of patients with histologically confirmed LAPC who underwent SBRT at Asan Medical Center from April 2011 to July 2016. Patients were excluded if they had: (1) metastatic disease at the time of SBRT, (2) prior abdominal radiotherapy, (3) other malignancies diagnosed within 5 years, or (4) gastric or duodenal invasion. This study was approved by the institutional review board of Asan Medical Center, and informed consent was waived due to the retrospective nature of the study.

Initial staging included physical examination, complete blood count, standard blood chemistry panel including carbohydrate antigen 19–9 (CA19-9), pancreatic protocol computed tomography (CT) scan, chest radiograph, magnetic resonance imaging (MRI), and positron emission tomography-CT (PET-CT) scan.

### SBRT procedure

In the majority of patients, three or four gold seeds were implanted into or near the pancreatic tumor using endoscopic ultrasound guidance a least one week prior to CT simulation. The stent was used an internal marker in patients who had endoscopic pancreatic duct stent placement. The SBRT procedure at our institution was described in our previous paper [17]. Briefly, four-dimensional CT (GE LightSpeed RT 16; GE Healthcare, Waukesha, WI, USA) simulation was performed during free breathing. A Real-time Position Management respiratory gating system (Varian Medical Systems, Palo Alto, CA, USA) was used to record the patients’ breathing patterns. The CT slice thickness was 2.5 mm and an intravenous contrast agent was injected to improve the accuracy of target and normal organ delineation. The CT data were sorted into ten CT series according to respiratory phase using 4D imaging software (Advantage 4D version 4.2; GE Healthcare).

Contouring and treatment planning were performed using a 3-dimensional radiotherapy planning system (Eclipse; Varian Medical Systems) based on the CT images at the end-expiratory phase. The gross tumor volume (GTV) was identified using diagnostic CT, MRI, and PET-CT images. The motion ranges of the GTV and normal organs were evaluated by examining all CT images from other phases. To reduce internal motion margins, a respiratory gating scheme was applied in all patients. After determining the adequate gating window around the end-expiratory phase (30 to 70% in most cases), the maximum intensity projection (MIP) images generated from the CT sets corresponding to the gating window were consulted to contour the internal target volume (ITV). To assess marker movement during simulation, marker trajectory was delineated using full-phase MIP images and registered on the end-expiratory phase CT images. Finally, the planning target volume (PTV) was defined using 3-mm isotropic margins to the ITV in order to account for set-up errors unless the margin resulted in expansion into the duodenum or stomach. In such cases, a nonuniform PTV margin expansion was used, provided that the GTV dose constraints were met. The metastatic regional lymph nodes were included in the target volume. Prescribed dose was administered to the isodose line covering the PTV. The total dose was mainly determined based on general dosing guidelines after determining the dose to be administered to the normal organs, including the following: maximal point dose to the stomach, duodenum, or small bowel was kept to < 30 Gy; ≥ 700 cm^3^ of normal liver was kept to < 15 Gy. The volume of 75% of combined kidneys was < 12 Gy, and maximal point dose to the spinal cord was < 20 Gy.

**Fig 1:**
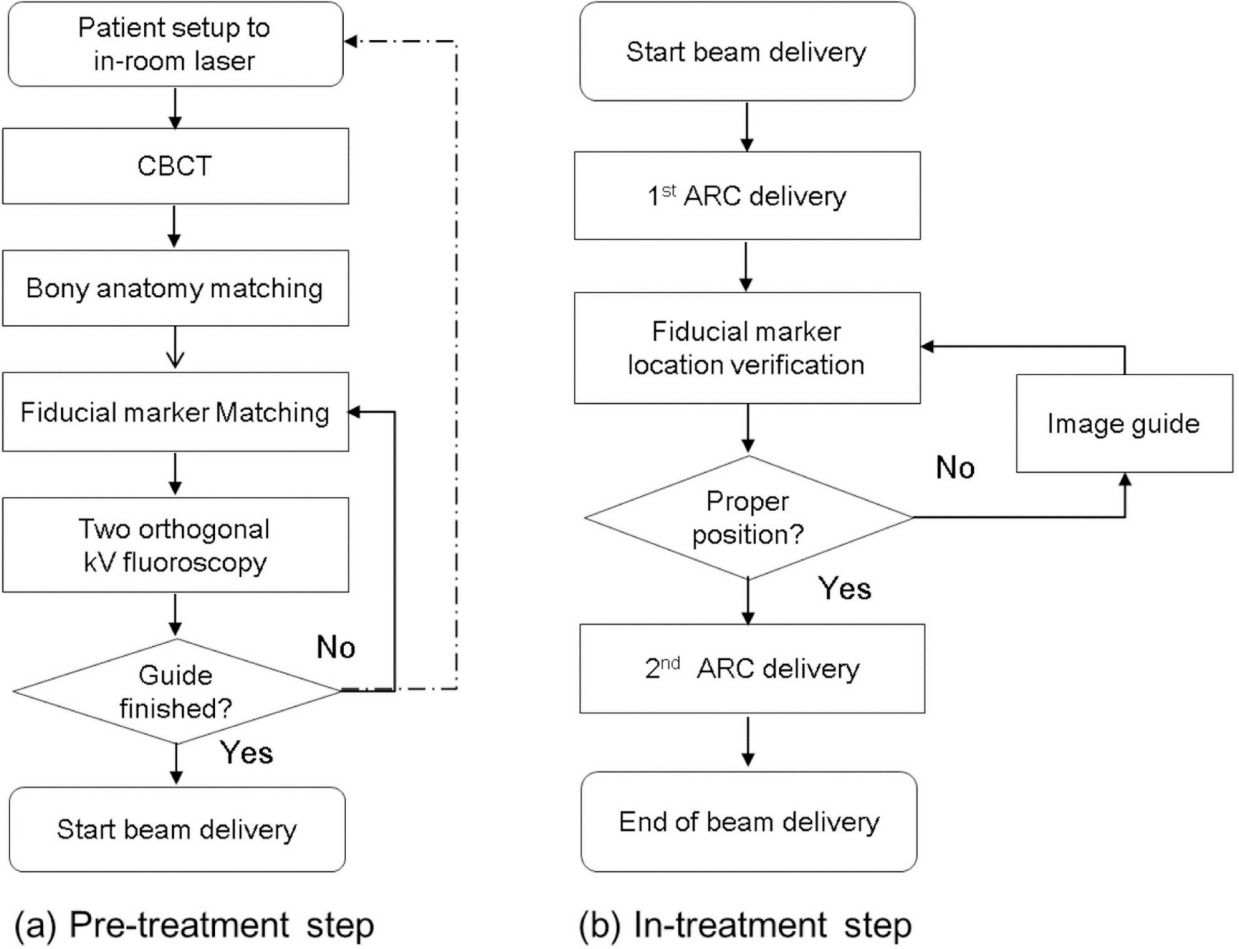
Protocol for marker-guided gated volumetric-modulated arc therapy. The protocol consists of two steps—pre-treatment and in-treatment. (a) Pre-treatment: patient alignment and image guidance are performed. If alignment is significantly out of range at each procedure, patient alignment is performed again. (b) In-treatment: after delivering the first arc dose, the half-time fiducial markers are verified.

During the initial period, a portion of patients (14, 14.7%) were treated with respiratory-gated IMRT; afterward, most patients (81, 85.3%) were treated with respiratory-gated VMAT. Fig 1 shows the overall procedure for marker-guided gated VMAT and SBRT [17]. At each treatment delivery, kV Cone Beam CT scans were performed to localize treatment targets through 4-dimensional registration (in 3D Cartesian coordinates and in-couch rotation angle) on the end-expiratory phase CT image. After registration, 2 orthogonal fluoroscopic kV images were acquired to confirm the respiratory motion of the fiducial markers. All markers were projected onto digitally reconstructed radiographs of the end-expiratory phase images. To minimize intra-fractional variation during treatment delivery, two-orthogonal fluoroscopic guidance was conducted halfway through beam delivery just after delivery of the first arc. All image guidance modalities were performed by a radiation oncologist and a medical physicist.

### Follow-up and statistics

All patients were examined during SBRT to assess acute toxicity. After treatment, regular follow-up examinations were performed at intervals of 2 to 3 months. Each patient’s prior medical history, physical examinations, complete blood counts, biochemical profiles, tumor markers, and imaging studies were reviewed at each follow-up. Induction chemotherapy was defined as the start of chemotherapy more than 1 month before SBRT. Adverse effects related to SBRT were graded according to the Common Terminology Criteria for Adverse Events, version 4.03. Acute toxicity was defined as adverse events occurring within 3 months after SBRT, and late toxicity was defined as those occurring after 3 months.

Overall survival (OS) and progression-free survival (PFS) were estimated from the date of diagnosis of pancreatic cancer to the date of death, the last follow-up examination, and to the date of any site of tumor progression, respectively. Local control (LC) was defined as absence of radiologic or clinical disease progression or recurrence within the treatment field. Freedom from local disease progression (FFLP) was calculated from the date of diagnosis to the date of local disease progression. The following factors were evaluated for their impact on the different survival endpoints: age, sex, Eastern Cooperative Oncology Group performance status, nodal metastasis, tumor size, tumor location, pre-SBRT CA19-9 level, post-SBRT CA19-9 level, induction chemotherapy, and SBRT dose. Equivalent dose in 2 Gy fractions (EQD2, using the linear-quadratic model, assuming an α/β of 10 Gy for pancreatic tumor) was used to compare between different fractionation schedules. The probability of cumulative survival was calculated using the Kaplan-Meier method, and was compared using the log-rank test. P values < 0.05 were considered statistically significant. Statistical analyses were performed using the Statistical Package for Social Science software, version 21 (IBM SPSS Statistics, Armonk, NY, USA).

## Results

### Patient characteristics

A total of 95 patients with histologically confirmed LAPC who underwent SBRT were included. Their characteristics are listed in Table 1. The median age at the time of diagnosis was 64 years (range, 38–84 years) and 51.6% were male. Fifty-two patients (54.7%) had pancreatic head cancers. A total of 90 patients (94.7%) received chemotherapy before or after SBRT. Induction chemotherapy was defined as chemotherapy for more than 1 month before SBRT, and 13 patients received induction chemotherapy which consisted of gemcitabine-based chemotherapy (n = 10) or modified FOLFIRINOX (n = 3). A total of 77 patients received chemotherapy within 1 month before SBRT or after SBRT. All these patients but 4 patients (modified FOLFIRINOX (n = 3) or FOLFIRI (n = 1)) received gemcitabine-based chemotherapy. A median dose of 28 Gy (range, 24–36 Gy) was given over 4 consecutive fractions delivered within 1 week, except in 3 patients who received 25 Gy in 5 fractions due to normal organ dose constraint. All patients completed treatment without interruption due to any reason during the SBRT course. There was no statistically significant difference in the prescription dose according to tumor location (head vs. body/tail, *p* = 0.853).

**Table 1:** Patient characteristics.

Characteristics		No. of patients (%, out of 95 patients)
Age	Median (range)	64 years (38–84 years)
Sex	Male	49 (51.6)
	Female	46 (48.4)
ECOP PS	0–1	88 (92.6)
	2	7 (7.4)
Nodal metastasis	No	72 (75.8)
	Yes	23 (24.2)
Tumor size	≤ 4 cm	32 (33.7)
	> 4 cm	63 (66.3)
Tumor location	Head	52 (54.7)
	Body/tail	43 (45.3)
Pre-SBRT CA19-9	≤ 200 U/mL	44 (46.3)
	> 200 U/mL	51 (53.7)
Sequential chemotherapy before or after SBRT	No	5 (5.3)7)
	Yes	90 (94.7)
Induction chemotherapy	No	82 (86.3)
	Yes	13 (13.7)
SBRT Dose	Median (range)	28.0 Gy (24–36 Gy)
Time from diagnosis to SBRT	Median (range)	19 days (8–282 days)

*Abbreviations*: ECOG PS = Eastern Cooperative Oncology Group performance status; SBRT = stereotactic body radiation therapy; CA19-9 = carbohydrate antigen 19–9.

### Treatment outcomes and prognostic factors

**Fig 2:**
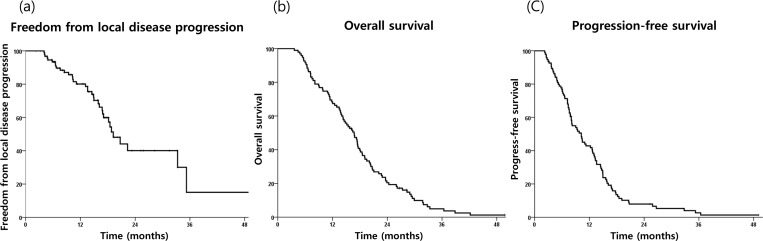
Kaplan-Meier curves of (a) freedom from local disease progression, (b) overall survival, and (c) progression-free survival in the study population.

Over a median follow-up period of 15 months (range, 2–49 months), median OS and PFS were 16.7 months and 10.2 months, respectively, and the 1-year OS and PFS rates were 67.4% and 42.9%, respectively (Fig 2). The 1-year FFLP rate was 80.1%. Among the 79 patients who experienced failure, the sites of first failures were isolated local progression in 12 patients (15.2%), DM in 55 patients (69.6%), and both in 12 patients (15.2%) (Table 2). Regional lymph node metastasis occurred in 7 patients, of these, 2 were lymph node metastasis without local progression or other DM. Seven patients (7.4%) were able to undergo surgical resection after SBRT and 4 patients had margin-negative resection.

**Table 2:** Patterns of failure (first site of recurrence).

Patterns	No. of patients (%, out of 79 patients who experienced failure)
Local	12 (15.2)
Distant	55 (69.6)
Local + Distant	12 (15.2)

As summarized in Table 3, absence of nodal metastasis was associated with favorable FFLP on univariate (1-year FFLP of 85.2% vs. 64.3%, *p* = 0.030) and multivariate analysis (*p* = 0.024). Post-SBRT CA19-9 ≤ 90 U/mL was associated with favorable OS and PFS on univariate (1-year OS of 81.6% vs. 53.7%, *p* = 0.013; 1-year PFS of 60.2% vs. 28.6%, *p* = 0.001) and multivariate analysis (OS, *p* = 0.014; PFS, *p* = 0.001). Tumor location (head vs. body/tail) or SBRT EQD_2_ (≤ 40 Gy_10_ vs. > 40 Gy_10_) did not have statistically significant effect on FFLP.

**Table 3:** Univariate analysis of covariates associated with FFLP, PFS, and OS.

variables		1-Y FFLP (%)	P-value	1-Y PFS (%)	P-value	1-Y OS (%)	P-value
Age	≤ 65	81.5	0.766	46.7	0.502	736	0.278
	> 65	77.9		37.9		61.9	
Sex	Male	75.5	0.260	43.5	0.330	65.3	0.123
	Female	84.6		42.3		69.6	
ECOG PS	0–1	80.2	0.299	43.6	0.717	70.5	0.218
	2	75.0		34.3		42.9	
Nodal metastasis	No	85.2	0.030*	47.1	0.176	66.7	0.338
	Yes	64.3		30.4		69.6	
Tumor size	≤ 4 cm	76.5	0.671	43.8	0.141	75.0	0.075
	> 4 cm	82.1		42.5		63.5	
Tumor location	Head	74.5	0.433	45.2	0.365	67.3	0.347
	Body-tail	86.8		40.3		67.4	
Pre-SBRT CA19-9	≤ 200 U/mL	77.5	0.101	49.5	0.191	75.0	0.224
	> 200 U/mL	82.9		37.1		60.8	
Post-SBRT CA19-9	≤ 90 U/mL	82.5	0.252	60.2	0.001*	81.6	0.013*
	> 90 U/mL	77.2		28.6		53.7	
Induction chemotherapy	No	79.8	0.924	44.0	0.382	65.9	0.177
	Yes	82.5		34.6		76.9	
SBRT EQD_2_	≤ 40 Gy_10_	81.7	0.763	44.6	0.822	66.7	0.408
	> 40 Gy_10_	78.3		40.8		68.2	

*Abbreviations*: FFLP = freedom from local disease progression; PFS = progression-free survival; OS = overall survival; ECOG PS = Eastern Cooperative Oncology Group performance status; SBRT = stereotactic body radiation therapy; CA19-9 = carbohydrate antigen 19–9; BED = biologically effective dose.

*statistically significant on multivariate analysis

### Toxicity

Three patients experienced grade 3 acute gastrointestinal (GI) toxicity (nausea and vomiting) during SBRT, which were mitigated with conservative management. Grade 3 late toxicity was observed in 3 (3.2%) patients. Two patients experienced duodenal ulcer bleeding and one patient experienced gastric ulcer perforation. Two patients who experienced duodenal ulcer bleeding were 49-year-old and 59-year-old females who received 30 Gy and 36 Gy in 4 fractions, respectively, to the head-body pancreatic cancer without duodenal invasion. At 5 and 6 months after the completion of SBRT, duodenal ulcer bleedings with tumor invasion were observed on endoscopic examination without active bleeding. The symptoms were mitigated with proton pump inhibitor. The GI bleeding is likely the effect of local tumor invasion, but could also be due to the late effect of SBRT. One patient who experienced gastric ulcer perforation after SBRT was a 52-year-old female who received 28 Gy in 4 fractions to the body-tail pancreatic cancer. Two months after completion of SBRT, the patient complained of epigastric discomfort and gastric ulcer perforation was detected on endoscopic examination. However, there was no evidence of tumor invasion to the stomach, and the distance from the irradiated field was quite far, so the relationship with the SBRT was not clear. The symptom was also mitigated with proton pump inhibitor. No patient died of treatment-related toxicities.

## Discussion

Several studies on conventional fractionated radiotherapy for LAPC showed that although the major patterns of treatment failure was DM, there were high rates of local progression that led to development of pancreatic pain, obstruction symptoms, and other morbidities that decrease the quality of life [5]. Therefore, improving local control is still an important aim of radiotherapy in LAPC patients. SBRT with single fraction for LAPC could give excellent FFLP: early SBRT studies for LAPC used single fraction radiotherapy and reported 1-year FFLP rates of 84 to 100% [7,8,12,14]. However, single-fraction SBRT was shown to result in significant late complication (grade 2 or above late toxicity of 13–47%). Recently, Herman et al. reported reduced incidence of late GI toxicity (grade 3 or above late toxicity of 6%) with fractionated SBRT of 33 Gy in 5 fractions compared with a historical cohort of patients treated with gemcitabine plus a single 25-Gy fraction SBRT [11]. Several studies of fractionated SBRT also showed favorable 1-year FFLP rates of 70–87% and minimal toxicities (Table 4) [10,11,13,15,16]. Considering equivalent dose in 2 Gy fractions (EQD_2_, using the linear-quadratic model, assuming an α/β of 10 Gy for pancreatic tumor), the prescribed dose of fractionated SBRT studies was similar to those of conventional CRT studies. However, fractionated SBRT showed comparable local control rate with single-fraction SBRT. In the present study, 1-year FFLP rate was 80.1%, which is in line with those of previous studies (Table 4). Furthermore, acute and late toxicities were low and similar to those of previous fractionated SBRT studies. All 95 patients received SBRT using a respiratory-gated VMAT or IMRT technique, and three patient experienced grade 3 acute GI toxicity (nausea and vomiting), while another 3 patients experienced grade 3 late toxicity (2 duodenal ulcer bleeding and 1 gastric ulcer perforation). All acute and late complications were mitigated with conservative management and no patient died of treatment-related toxicities. Considering the poor prognosis of LAPC patients, our dose-fraction scheme of 28 Gy in 4 fractions seems to be a reasonable option regarding local control and toxicity.

**Table 4:** Comparison of clinical outcomes from previous studies of fractionated SBRT for locally advanced pancreatic cancer.

Study	Scheme	Dose	EQD_2_	N	1Y-FFLP (%)	Median OS	Acute toxicity grade ≥ 3	Late GI toxicity grade ≥ 3	Dose constraints for GI tract
Hoyer[9]	SBRT alone	45 Gy/3fx	94 Gy_10_	22	57% (6months)	5.4	79% (grade ≥ 2)	23%	-
Polistina[10]	SBRT + CTx	30 Gy/3fx	50 Gy_10_	23	70% (crude)	10.6	0%	0%	-
Mahadevan[16]	SBRT + CTx	24 Gy/3fx (24–30 Gy)	36 Gy_10_ (36–50 Gy_10)_	39	85% (crude)	20.0	0%	8%	D_max_ of bowel < 30 Gy
Herman[11]	SBRT + CTx	33 Gy/5fx	46 Gy_10_	49	78%	13.9	12%	6%	D_1 cm3_ of duodenum and stomach < 33 Gy
Comito[13]	SBRT + CTx	45 Gy/6fx	66 Gy_10_	45	87%	13.0	0%	0%	D_1 cm3_ of duodenum < 36 Gy, D_3 cm3_ of stomach < 36 Gy
Mazzola[15]	SBRT + CTx	36 Gy/6fx (36–45 Gy)	48 Gy_10_ (48–66 Gy_10_)	33	81%	75% (1 year)	0%	0%	D_1 cm3_ of duodenum < 36 Gy, D_3 cm3_ of stomach < 36 Gy
Current study	SBRT + CTx	28 Gy/4fx (24–36 Gy)	40 Gy_10_ (32–57 Gy_10_)	95	80%	16.7	3%	3%	D_max_ of duodenum and stomach < 30 Gy

*Abbreviations*: SBRT = stereotactic body radiation therapy; EQD_2_ = Equivalent dose in 2 Gy fractions; N = number; FFLP = freedom from local disease progression; OS = overall survival; GI = gastrointestinal; CTx = chemotherapy; fx = fraction; D_max_ = maximum dose; D_x_ = dose per x cc.

It is difficult to obtain significant survival benefit by improving local control in patients with LAPC because the major pattern of progression is DM. Our present study also showed that the major patterns of failure were DM and local control did not significantly affect survival (data not reported). However, the median OS was 16.7 months in this study, which was a relatively favorable survival outcome compared with 7.6–15.2 months median OS from historical studies that used conventional CRT [18–23]. Two studies from National Cancer Data Base in the US also showed that SBRT was associated with superior overall survival than conventional CRT [24,25]. SBRT without delaying for intensive chemotherapy could a possible reason. In our study, the majority of patients (94.7%) received gemcitabine-based chemotherapy. Prior fractionated SBRT studies also reported improved survival (median OS 10.6–20.0 months) without delaying for intensive chemotherapy. Herman et al. showed that fractionated SBRT with gemcitabine results in minimal acute and late GI toxicity [11]; the authors conducted a single-arm, phase 2 study to determine whether LAPC patients treated with gemcitabine administered with fractionated SBRT of 33 Gy in 5 fractions would achieve reduced late GI toxicity compared with single 25-Gy fraction SBRT. A total of 49 patients received up to 3 doses of gemcitabine followed by a 1-week break and SBRT. After SBRT, patients could continue to receive gemcitabine of median number of 7 doses until disease progression or toxicity. They reported 12.2% incidence of acute grade ≥ 3 toxicity and 6% of late grade ≥ 3 toxicity, and a median OS of 13.9 months. Gurka et al. also reported that SBRT with concurrent full-dose gemcitabine was safe when administered to LAPC patients [26]. In their study, patients received gemcitabine for 6 cycles, and during week 4 of cycle 1, patients received fractionated SBRT of 25 Gy in 5 fractions. There were no grade 3 or greater radiation-related toxicities or delays for cycle 2 of gemcitabine.

The optimal scheme of chemotherapy and SBRT has not been established, for which further studies are needed. However, it may be possible to introduce a useful therapeutic strategy from the LAP-07 trial, which was conducted to assess whether CRT improves OS in patients with LAPC that was controlled after 4 months of gemcitabine-based induction chemotherapy [23]. The LAP-07 trial applied a therapeutic strategy that selects patients who could potentially benefit from CRT after induction chemotherapy, OS were higher in patients who received CRT (15.2 months) or maintenance chemotherapy (16.5 months) than that (7.7 months) in patients who were excluded from the trial due to disease progression, poor performance status, or severe toxic effects during induction chemotherapy. It is expected that optimal treatment scheme of chemotherapy and fractionated SBRT through further research could result in a more effective combined modality strategy and subsequent survival benefit.

Dose-response relationship between fractionated SBRT and pancreatic cancer is an important yet unresolved issue. In our present study, there was no significant correlation between 1-year FFLP rate and SBRT EQD_2_ (≤ 40 Gy_10_ vs. > 40 Gy_10_), which was difficult to define from previous studies as well as our current study due to similar SBRT dose and small number of patients. There is an ongoing study for evaluating the efficacy of higher SBRT dose using MRI-guided adaptive radiotherapy to minimize toxicity; the results of this study may show whether higher SBRT dose leads to significantly better local control and subsequent overall survival benefit.

The present study had certain limitations of note. The systemic therapy used in the current study could be inferior to current practice that uses a more effective regimen such as FOLFIRINOX and gemcitabine plus Abraxane. The effect of local modality may be mitigated with improved systemic control, and future SBRT trials with the improved regimens would provide more information on this issue. Also, because the overall survival in this cohort was not sufficiently long to address long term effects on local control, 2-year FFLP may be a more desirable endpoint as compared with 1-year FFLP in the setting of better systemic control. There is a need for future trials regarding better local control as systemic therapy improved. Nevertheless, the current analysis had several unique strengths: 1) this study included the large number of patients from a single center who received SBRT for locally advanced unresectable pancreatic cancer; 2) the entire study cohort homogeneously received advanced SBRT technique using a respiratory-gated VMAT or IMRT; and 3) the various patient conditions and treatment situations can be considered to well-reflect real-life practice.

In conclusion, chemotherapy and SBRT with a median dose of 28 Gy in 4 fractions in LAPC patients resulted in favorable FFLP and OS with minimal treatment-related toxicity. Considering that the most common pattern of failure was DM, future studies should focus on developing the optimal scheme of chemotherapy and SBRT for patients with LAPC.

## Supporting information

S1 Dataset (XLSX)

## References

[1] Timmerman RD, Kavanagh BD, Cho LC, Papiez L, Xing L. Stereotactic body radiation therapy in multiple organ sites. J Clin Oncol. 2007; 25: 947–952. 10.1200/JCO.2006.09.7469 17350943

[2] Timmerman R, Paulus R, Galvin J, Michalski J, Straube W, Bradley J, et al Stereotactic body radiation therapy for inoperable early stage lung cancer. JAMA. 2010; 303: 1070–1076. 10.1001/jama.2010.261 20233825 PMC2907644

[3] Hidalgo M. Pancreatic cancer. N Engl J Med. 2010; 362: 1605–1617. 10.1056/NEJMra0901557 20427809

[4] Balaban EP, Mangu PB, Khorana AA, Shah MA, Mukherjee S, Crane CH, et al Locally advanced, unresectable pancreatic cancer: American society of clinical oncology clinical practice guideline. J Clin Oncol. 2016; 34: 2654–2668. 10.1200/JCO.2016.67.5561 27247216

[5] Willett CG, Czito BG, Bendell JC, Ryan DP. Locally advanced pancreatic cancer. J Clin Oncol. 2005; 23: 4538–4544. 10.1200/JCO.2005.23.911 16002845

[6] Mahadevan A, Jain S, Goldstein M, Miksad R, Pleskow D, Sawhney M, et al Stereotactic body radiotherapy and gemcitabine for locally advanced pancreatic cancer. Int J Radiat Oncol Biol Phys. 2010; 78: 735–742. 10.1016/j.ijrobp.2009.08.046 20171803

[7] Schellenberg D, Goodman KA, Lee F, Chang S, Kuo T, Ford JM, et al Gemcitabine chemotherapy and single-fraction stereotactic body radiotherapy for locally advanced pancreatic cancer. Int J Radiat Oncol Biol Phys. 2008; 72: 678–686. 10.1016/j.ijrobp.2008.01.051 18395362

[8] Chang DT, Schellenberg D, Shen J, Kim J, Goodman KA, Fisher GA, et al Stereotactic radiotherapy for unresectable adenocarcinoma of the pancreas. Cancer. 2009; 115: 665–672. 10.1002/cncr.24059 19117351

[9] Hoyer M, Roed H, Sengelov L, Traberg A, Ohlhuis L, Pedersen J, et al Phase-II study on stereotactic radiotherapy of locally advanced pancreatic carcinoma. Radiother Oncol. 2005; 76: 48–53. 10.1016/j.radonc.2004.12.022 15990186

[10] Polistina F, Costantin G, Casamassima F, Francescon P, Guglielmi R, Panizzoni G, et al Unresectable locally advanced pancreatic cancer: a multimodal treatment using neoadjuvant chemoradiotherapy (gemcitabine plus stereotactic radiosurgery) and subsequent surgical exploration. Ann Surg Oncol. 2010; 17: 2092–2101. 10.1245/s10434-010-1019-y 20224860

[11] Herman JM, Chang DT, Goodman KA, Dholakia AS, Raman SP, Hacker-Prietz A, et al Phase 2 multi-institutional trial evaluating gemcitabine and stereotactic body radiotherapy for patients with locally advanced unresectable pancreatic adenocarcinoma. Cancer. 2015; 121: 1128–1137. 10.1002/cncr.29161 25538019 PMC4368473

[12] Schellenberg D, Kim J, Christman-Skieller C, Chun CL, Columbo LA, Ford JM, et al Single-fraction stereotactic body radiation therapy and sequential gemcitabine for the treatment of locally advanced pancreatic cancer. Int J Radiat Oncol Biol Phys. 2011; 81: 181–188. 10.1016/j.ijrobp.2010.05.006 21549517

[13] Comito T, Cozzi L, Clerici E, Franzese C, Tozzi A, Iftode C, et al Can stereotactic body radiation therapy be a viable and efficient therapeutic option for unresectable locally advanced pancreatic adenocarcinoma? Results of a phase 2 study. Technol Cancer Res Treat. 2017; 16: 295–301. 10.1177/1533034616650778 27311310 PMC5616043

[14] Koong AC, Le QT, Ho A, Fong B, Fisher G, Cho C, et al Phase I study of stereotactic radiosurgery in patients with locally advanced pancreatic cancer. Int J Radiat Oncol Biol Phys. 2004; 58: 1017–1021. 10.1016/j.ijrobp.2003.11.004 15001240

[15] Mazzola R, Fersino S, Aiello D, Gregucci F, Tebano U, Corradini S, et al Linac-based stereotactic body radiation therapy for unresectable locally advanced pancreatic cancer: risk-adapted dose prescription and image-guided delivery. Strahlenther Onkol. 2018; 194: 835–842. 10.1007/s00066-018-1306-2 29696321

[16] Mahadevan A, Miksad R, Goldstein M, Sullivan R, Bullock A, Buchbinder E, et al Induction gemcitabine and stereotactic body radiotherapy for locally advanced nonmetastatic pancreas cancer. Int J Radiat Oncol Biol Phys. 2011; 81: e615–622. 10.1016/j.ijrobp.2011.04.045 21658854

[17] Cho I, Park JW, Cho B, Kwak J, Yoon SM, Nesseler JP, et al Dosimetric analysis of stereotactic rotational versus static intensity-modulated radiation therapy for pancreatic cancer. Cancer Radiother. 2018; 22: 754–762. 10.1016/j.canrad.2018.01.007 30322818

[18] Moertel CG, Childs DS Jr., Reitemeier RJ, Colby MY Jr., Holbrook MA. Combined 5-fluorouracil and supervoltage radiation therapy of locally unresectable gastrointestinal cancer. Lancet. 1969; 2: 865–867. 10.1016/S0140-6736(69)92326-5 4186452

[19] Gastrointestinal Tumor Study Group. Radiation therapy combined with Adriamycin or 5-fluorouracil for the treatment of locally unresectable pancreatic carcinoma. Cancer. 1985; 56: 2563–2568. 10.1002/1097-0142(19851201)56:11<2563::AID-CNCR2820561104>3.0.CO;2-0 2864997

[20] Klaassen DJ, MacIntyre JM, Catton GE, Engstrom PF, Moertel CG. Treatment of locally unresectable cancer of the stomach and pancreas: a randomized comparison of 5-fluorouracil alone with radiation plus concurrent and maintenance 5-fluorouracil—an Eastern Cooperative Oncology Group study. J Clin Oncol. 1985; 3: 373–378. 10.1200/JCO.1985.3.3.373 3973648

[21] Chauffert B, Mornex F, Bonnetain F, Rougier P, Mariette C, Bouche O, et al Phase III trial comparing intensive induction chemoradiotherapy (60 Gy, infusional 5-FU and intermittent cisplatin) followed by maintenance gemcitabine with gemcitabine alone for locally advanced unresectable pancreatic cancer. Definitive results of the 2000–01 FFCD/SFRO study. Ann Oncol. 2008; 19: 1592–1599. 10.1093/annonc/mdn281 18467316

[22] Loehrer PJ Sr., Feng Y, Cardenes H, Wagner L, Brell JM, Cella D, et al Gemcitabine alone versus gemcitabine plus radiotherapy in patients with locally advanced pancreatic cancer: an Eastern Cooperative Oncology Group trial. J Clin Oncol. 2011; 29: 4105–4112. 10.1200/JCO.2011.34.8904 21969502 PMC3525836

[23] Hammel P, Huguet F, van Laethem JL, Goldstein D, Glimelius B, Artru P, et al Effect of chemoradiotherapy vs chemotherapy on survival in patients with locally advanced pancreatic cancer controlled after 4 months of gemcitabine with or without erlotinib: The LAP07 randomized clinical trial. JAMA. 2016; 315: 1844–1853. 10.1001/jama.2016.4324 27139057

[24] de Geus SWL, Eskander MF, Kasumova GG, Ng SC, Kent TS, Mancias JD, et al Stereotactic body radiotherapy for unresected pancreatic cancer: A nationwide review. Cancer. 2017; 123: 4158–4167. 10.1002/cncr.30856 28708929

[25] Zhong J, Patel K, Switchenko J, Cassidy RJ, Hall WA, Gillespie T, et al Outcomes for patients with locally advanced pancreatic adenocarcinoma treated with stereotactic body radiation therapy versus conventionally fractionated radiation. Cancer. 2017; 123: 3486–3493. 10.1002/cncr.30706 28493288 PMC5589506

[26] Gurka MK, Collins SP, Slack R, Tse G, Charabaty A, Ley L, et al Stereotactic body radiation therapy with concurrent full-dose gemcitabine for locally advanced pancreatic cancer: a pilot trial demonstrating safety. Radiat Oncol. 2013; 8: 44 10.1186/1748-717X-8-44 23452509 PMC3607991

